# Supplementary material for: In situ characterization of crystallization and melting of soft, thermoresponsive microgels by small-angle X-ray scattering
Source: Soft Matter. 2021 Dec 20;18(8):1591–602. doi: 10.1039/d1sm01537k (PMC8864529; doi:10.1039/d1sm01537k)
Supplement: SM-018-D1SM01537K-s001 [file SM-018-D1SM01537K-s001.pdf]

Electronic Supplementary Material (ESI) for Soft Matter.
This journal is © The Royal Society of Chemistry 2021

In-situ Characterization of Crystallization and Melting of Soft Thermoresponsive Microgels by Small-Angle X-ray Scattering

Supplementary Information

Dmitry Lapkin[1], Nastasia Mukharamova[1], Dameli Assalauova[1], Svetlana Dubinina[1,2], Jens Stellhorn[1,3], Fabian Westermeier[1], Sergey Lazarev[1,4], Michael Sprung[1], Matthias Karg[5,*], Ivan A. Vartanyants[1, 6,*], and Janne-Mieke Meijer[7,*]

[1]Deutsches-Elektronen Synchrotron DESY, Notkestrasse 85, 22607 Hamburg, Germany
[2]Moscow Institute of Physics and Technology (State University), Institutskiy Per. 9, 141701 Dolgoprudny, Moscow Region, Russia
[3]Department of Applied Chemistry, Graduate School of Advanced Science and Engineering, Hiroshima University, 1-4-1 Kagamiyama, Higashihiroshima 739-8527, Japan
[4]National Research Tomsk Polytechnic University (TPU), Lenin Avenue 30, 634050 Tomsk, Russia
[5]Heinrich-Heine-Universität Düsseldorf, Universitätsstraße 1, D-40225 Düsseldorf, Germany
[6]National Research Nuclear University MEPhI (Moscow Engineering Physics Institute), Kashirskoe shosse 31, 115409 Moscow, Russia
[7]Department of Applied Physics and Institute for Complex Molecular Systems, Eindhoven University of Technology, Groene Loper 19, 5612 AP Eindhoven, The Netherlands

[*]Corresponding authors: ivan.vartaniants@desy.de, j.m.meijer@tue.nl

S1. Core-shell particle characterization

The size of the gold core of the core-shell (CS) microgel particles was determined with Transmission Electron Microscopy (TEM, FEI Tecnai 20 (type Sphera)). Fig. S1a shows a representative TEM images of the core-shell particles, where the dense gold core (black centres) can be clearly distinguished from the hydrogel shell (light grey corona). Note that due to drying effects, the shells have collapsed into a pancake shape resulting in different sizes and the gold core to appear off-centre. Some of the microgel particles were found to not contain a gold core but these constitute less than 0.1% of all particles. Using ImageJ particle analysis the gold core size was determined from the area of the cores in the TEM images for more than 1000 particles, assuming a spherical shape (Fig. S1b). The average gold core radius was found to be R_{core} = 29.1 ± 4.2 nm.

The size of the CS microgels dispersed in water over a temperature range of T = 15 – 50 °C was determined from a 0.1 wt. % dispersion with dynamic light scattering (DLS) (Litesizer 500, Anton Paar, 175°, λ = 660 nm) with 3 measurements of 60 s and cumulant analysis fit. Fig. S1c shows the hydrodynamic radius R_h found for each temperature. The CS microgels decrease in size from R_h = 236.6 nm at 15 °C to R_h = 151.1 nm at 50 °C, which indicates a 75% volume decrease over the full temperature range. With a sigmoidal fit the VPTT transition was found at T = 32.3 °C, which is in agreement with typical VPTT values for aqueous PNIPAM microgels.[1] In addition, the zeta-potential ζ was determined from electrophoretic measurements. Fig. S1d shows ζ over the full temperature range and indicates that ζ decreases from ζ ~ -10 mV to ζ ~ -30 mV as the temperature is raised from T = 20 °C to T = 40 °C caused by the PNIPAM shell collapse and increase of surface charges.

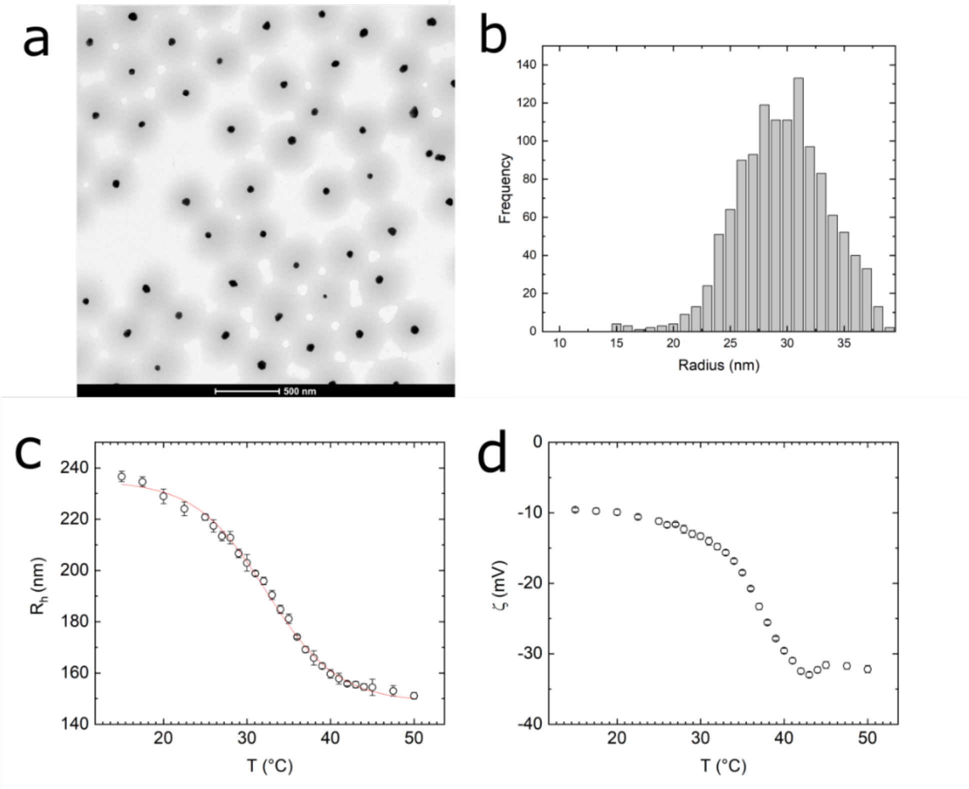

Figure S1. (**a**) Representative TEM images of the core-shell microgels with a gold core and hydrogel shell. (**b**) Size distribution of R_{core}. (**c**) Hydrodynamic radius R_h with sigmoidal fit (red line) and (**d**) the zeta-potential of the CS microgels in the range of T = 15 – 50 °C. Error bars represent measured polydispersity index (c) and standard deviation (d).

S2. Volume fraction determination

Particle dispersions were prepared at 0.5 wt% and 12 wt%. The temperature dependent volume fractions ϕ_{eff} (T) were determined from the number density, N, based on interparticle distance, a, extracted from the Bragg peak position in the SAXS patterns in the full crystallized state of the 12 wt% sample and the CS particle volume. For this we assume an overall FCC lattice and base the particle volume on the hydrodynamic radius, R_h(T), as measured with the DLS (Fig. S1c). The effective volume fraction is calculated according to:

$$\phi_{eff}(\mathrm{T}) = NV_{CS} = (V_{FCC}/4)V_{CS}(\mathrm{T}) = \left(\left(2a/\sqrt{2}\right)^3/4\right)(4/3\,\pi R_h(\mathrm{T})^3), \quad \text{(S1)}$$

where V_{cs}(T) is the CS particle volume controlled by temperature, and V_{FCC} is the *fcc* unit cell volume. At T = 38 °C we find a = 490 nm and R_h = 165.8 nm, resulting in ϕ_{eff} = 0.23. Table S1 shows an overview of the changing ϕ_{eff} with temperature based on this calculation. This shows that at T = 20 °C with R_h = 228.9 nm the system has a volume fraction of ϕ_{eff} = 0.60. By correcting for wt% we can extract ϕ_{eff} for the 0.5 wt% sample resultin in ϕ_{eff} = 0.025 at T = 20 °C and ϕ_{eff} = 0.010 at T = 38 °C.

S3. Temperature controlled sample holder

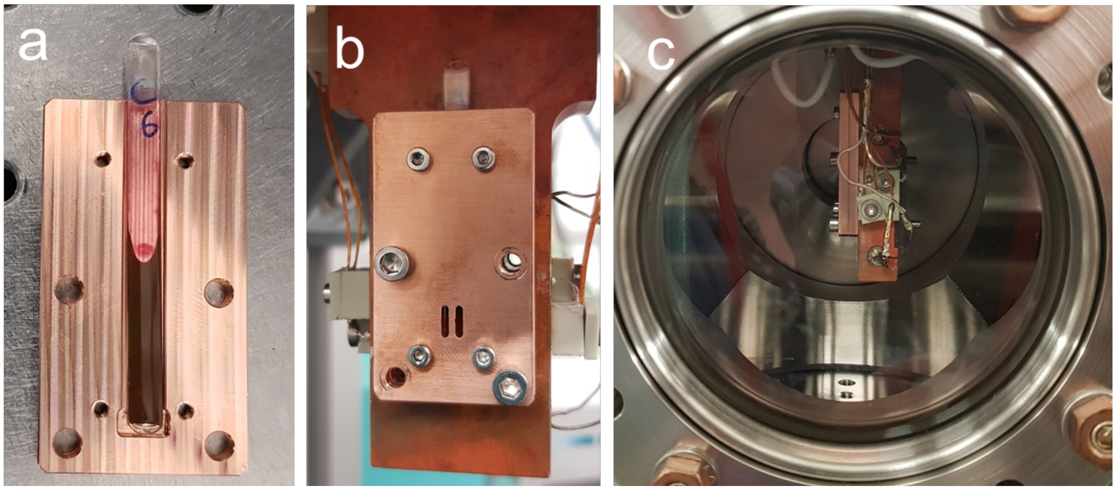

Figure S2 . Photographs of (**a**) one part of a copper sample holder that had an cavity into which the flame sealed capillary (4 × 0.2 × 50 mm^3, internal dimensions, Vitrocom) with CS particle dispersion fitted tightly, (**b**) the full copper sample holder with capillary mounted onto the Peltier element, (**c**) side view of sample holder in vacuum tube.

S4. Scattering from the dilute sample

We investigated the CS microgels in the dilute state with a concentration of 0.5 wt. % corresponding to a volume fraction ϕ_{eff}(20 °C) = 0.025. Measurements were performed in the temperature range of 25.0 °C < T < 50.0 °C. Details on the Ultra-Small Angle X-ray Scattering are given in Experimental section of the main text.

The theoretical description of the X-ray scattering is given according to the literature.[2,3] The scattered intensity in kinematical approximation from a dilute sample, where interference between scattering from different particles can be neglected (i.e. the structure factor S(q) ≈ 1) can be described as[2]

$$I(q) = N\Delta\rho^2 V^2 P(q)\,, \tag{S2}$$

where N is the number of illuminated particles in the scattering volume, $\Delta\rho = \rho - \rho_{solvent}$ is the scattering contrast (the electron density difference between the particles and medium (solvent)), V is the volume and $P(q) = |F(q)|^2$ the form factor of a single microgel.

The form factor amplitude for a homogeneous sphere with the radius R is[2]

$$F_1(q,R) = \frac{3[\sin(qR) - qR\cos(qR)]}{(qR)^3}, \tag{S3}$$

The polydispersity of the particles can be introduced by averaging over the particle radius distribution. For the normal size distribution, the probability density is

$$D(R,\langle R\rangle,\sigma_R) = \frac{1}{\sqrt{2\pi\sigma_R^2}}\exp\left[-\frac{(R-\langle R\rangle)^2}{2\sigma_R^2}\right]. \tag{S4}$$

where $<R>$ is the mean radius and σ_R is the standard deviation. Then, the scattered intensity can be expressed as[3]

$$I(q) = N\Delta\rho^2 \int_0^\infty D(R)V^2(R)|F_1(q,R)|^2 dR\,. \tag{S5}$$

The form factor amplitude for a core-shell particle can be obtained by a proper weighting of the partial amplitudes from the spherical core and shell as[3]

$$F_2(q,R_{core},R_{shell}) == \frac{(\Delta\rho_{core} - \Delta\rho_{shell})V(R_{core})F_1(q,R_{core}) + \Delta\rho_{shell}V(R_{shell})F_1(q,R_{shell})}{(\Delta\rho_{core} - \Delta\rho_{shell})V(R_{core}) + \Delta\rho_{shell}V(R_{shell})}, \tag{S6}$$

where R_{core} and R_{shell} are the radii, and $\Delta\rho_{core}$ and $\Delta\rho_{shell}$ are the scattering contrasts of the core and shell, respectively. The particle volume V(R) in this case is equal to the sphere volume $V(R) = \frac{4}{3}\pi R^3$.

By analogy, the polydispersity can be taken into account if one considers the core and shell radii distributions. Then, the resulting scattered intensity can be defined as

$$\begin{aligned} I(q) = N\int_0^\infty\int_0^\infty D\big(R_{core},\langle R_{core}\rangle,\sigma_{R_{core}}\big)D\big(R_{shell},\langle R_{shell}\rangle,\sigma_{R_{shell}}\big) \times \\ \times [(\Delta\rho_{core} - \Delta\rho_{shell})V(R_{core}) + \Delta\rho_{shell}V(R_{shell})]^2|F_2(q,R_{core},R_{shell})|^2 dR_{core}dR_{shell} \end{aligned} \tag{S7}$$

where $\langle R_{core}\rangle$ and $\langle R_{shell}\rangle$ are the mean radii and $\sigma_{R_{core}}$ and $\sigma_{R_{shell}}$ are the standard deviation of the radii of the core and shell, respectively.

We define an effective form factor for polydisperse core-shell particles as

$$\begin{aligned} P_{cs}(q) = \frac{1}{\langle(\Delta\rho V)^2\rangle}\int_0^\infty\int_0^\infty D\big(R_{core},\langle R_{core}\rangle,\sigma_{R_{core}}\big)D\big(R_{shell},\langle R_{shell}\rangle,\sigma_{R_{shell}}\big) \times \\ \times [(\Delta\rho_{core} - \Delta\rho_{shell})V(R_{core}) + \Delta\rho_{shell}V(R_{shell})]^2|F_2(q,R_{core},R_{shell})|^2 dR_{core}dR_{shell} \end{aligned} \tag{S8}$$

where

$$\langle(\Delta\rho V)^2\rangle = \int_0^\infty \int_0^\infty D\left(R_{core}, \langle R_{core}\rangle, \sigma_{R_{core}}\right) D\left(R_{shell}, \langle R_{shell}\rangle, \sigma_{R_{shell}}\right) \times \\ \times \left[(\Delta\rho_{core} - \Delta\rho_{shell})V(R_{core}) + \Delta\rho_{shell}V(R_{shell})\right]^2 dR_{core}dR_{shell}. \quad \text{(S9)}$$

We fitted the radially-averaged intensity profiles of the diffraction patterns from the dilute samples shown in Fig. S2a,b. We used the polydisperse core-shell form factor given in Eq. S7 multiplied by the scaling parameter I_0:

$$I(q) = I_0 P_{cs}(q) \quad \text{(S10)}$$

The scattering contrast values were calculated in respect to water with the electron density ρ_{H_2O} = 335 nm^{-3}. The gold core electron density was considered to be ρ_{Au} = 4661 nm^{-3}. This gives a core scattering contrast of $\Delta\rho_{core}$ = 4326 nm^{-3} which was fixed in the fitting procedure.[4] All other parameters, namely, the scaling parameter I_0, the shell scattering contrast $\Delta\rho_{shell}$, the mean core ($\langle R_{core}\rangle$) and shell radii ($\langle R_{shell}\rangle$) and their standard deviations $\sigma_{R_{core}}$ and $\sigma_{R_{shell}}$, respectively, were used as fitting parameters to fit the intensity profiles at different temperatures T. Two examples of the fit at two characteristic temperatures of T = 25.0 °C and T = 50.0 °C are shown in Fig. S2c,d. By that, we extracted these parameters for the dilute sample measured at different temperatures in the range of T = 25.0 – 50.0 °C. The extracted parameters are shown in Fig. S3.

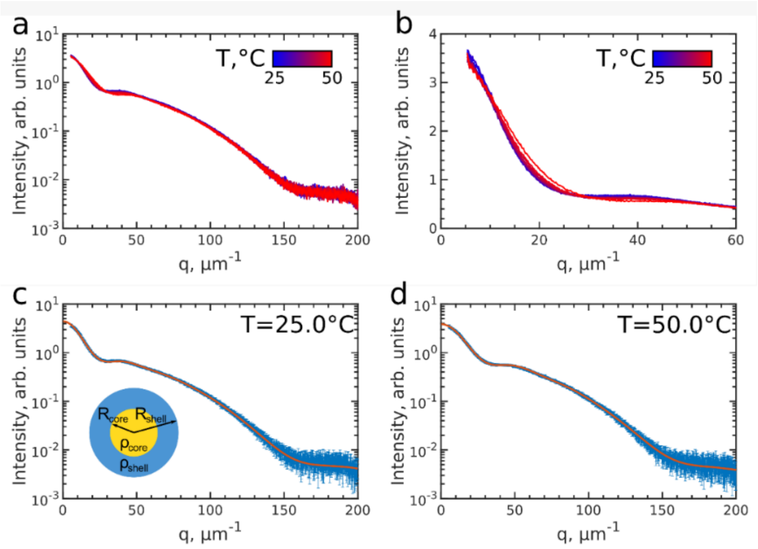

Figure S3. **(a)** Radially averaged intensity from the dilute sample at different temperatures in the range of T = 25.0 – 50.0 °C and **(b)** magnification of low-*q* area showing the change in the shell scattering. The error bars are omitted for clarity. **(c,d)** Examples of the measured intensities (blue lines) and the best fits with the core-shell model. for the two extreme temperatures: (c) T = 25.0 °C and (d) T = 50.0 °C. Inset in (c) shows the model parameters used for fitting.

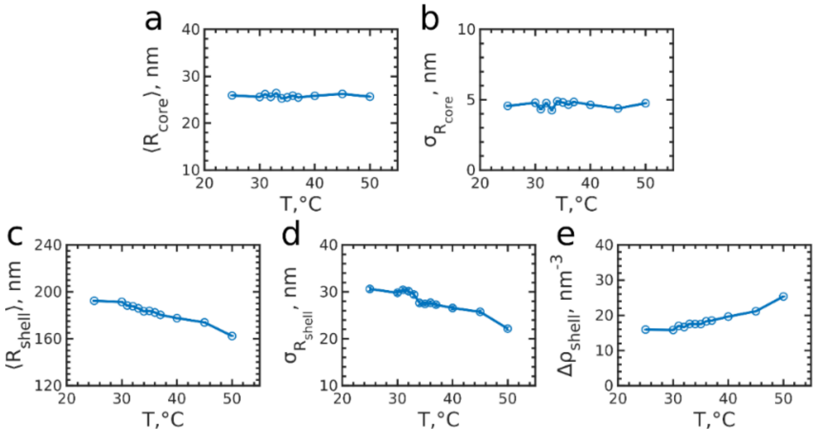

Figure S4. Evolution of the extracted parameters for the core-shell microgels with the temperature. **(a)** The mean gold core radius $\langle R_{core}\rangle$ and **(b)** its standard deviation $\sigma_{R_{core}}$, **(c)** mean shell radius $\langle R_{shell}\rangle$, **(d)** its standard deviation $\sigma_{R_{shell}}$, and **(e)** shell scattering contrast $\Delta\rho_{shell}$.

S5. Fluid structure factor

We now turn to the densely packed microgel sample with the concentration of 12 wt. % (an effective volume fraction ϕ_{eff}(20 °C) = 0.60). Due to the volume phase transition behaviour of the microgels, this sample will possess a significantly reduced effective volume fraction at high temperatures. Here, the sample reveals a typical fluid-like intensity profile, as shown in the main text in Fig. 3d and Fig. 6d. One of the analytically calculated structure factor models for a fluid is a hard-sphere model in the Percus-Yevick approximation.[3] In this model, the particles interact with the hard-sphere radius R_{PY} and have a hard-sphere volume fraction ϕ_{PY}. Then, the structure factor can be expressed as

$$S(q) = \frac{1}{1 + 24\phi_{PY}\frac{G(2qR_{PY})}{2qR_{PY}}}, \tag{S11}$$

where

$$G(x) = \alpha\frac{\sin x - x\cos x}{x^2} + \beta\frac{2x\sin x + (2 - x^2)\cos x - 2}{x^3} + \gamma\frac{-x^4\cos x + 4[(3x^2 - 6)\cos x + (x^3 - 6x)\sin x + 6]}{x^5}, \tag{S12}$$

and

$$\alpha = \frac{(1 + 2\phi_{PY})^2}{(1 - \phi_{PY})^4}, \quad \beta = \frac{-6\phi_{PY}(1 + \phi_{PY}/2)^2}{(1 - \phi_{PY})^4}, \quad \gamma = \alpha\phi_{PY}/2. \tag{S13}$$

The experimentally obtained 1D intensity profiles were fitted by this model using the hard sphere radius R_{PY} and the volume fraction ϕ_{PY} as the fitting parameters. The results of the fit are shown in the main text in Fig. 3d and Fig. 6d.

S6. Bragg peak fitting

An example of the diffraction pattern at T = 35 °C is shown in Fig. S4a. The pattern was interpolated into polar (q,φ)-coordinates and divided by the form factor of the CS nanoparticles $P_{cs}(q)$ extracted from the diluted sample as described in Section S2. At each q-value, the intensity between the Bragg peaks $S_{iso}(q)$ was calculated and additionally subtracted from the pattern to keep only the anisotropic Bragg peaks. The resulting pattern in polar coordinates is shown in Fig. S4b.

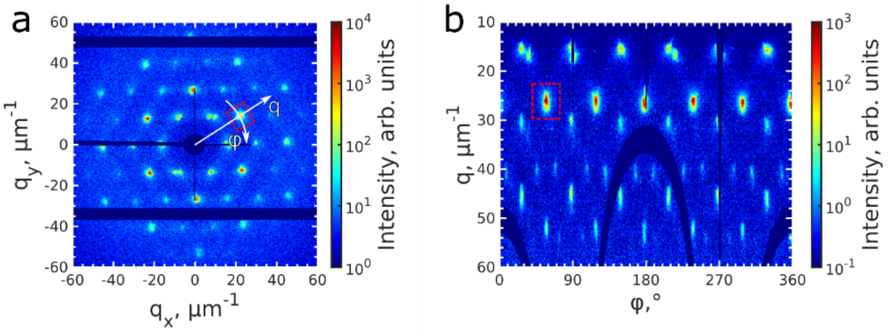

Figure S5. (a) Example of the diffraction patterns at T = 35 °C, when the sample is crystallized. **(b)** The same pattern interpolated into polar coordinates after division by the form factor and subtraction of the isotropic part. The highlighted peak is shown enlarged in Fig. S5a.

The peak highlighted in Fig. S4 is shown separately in Fig. S5a. It was fitted by the 2D Gaussian function:

$$G(q,\varphi) = \frac{I}{2\pi\sigma_q\sigma_\varphi}\exp\left[-\frac{(q-q_0)^2}{2\sigma_q^2} - \frac{(\varphi-\varphi_0)^2}{2\sigma_\varphi^2}\right], \tag{S8}$$

where I is the integrated intensity, q_0 and φ_0 are the peak positions in radial and azimuthal directions, and σ_q and σ_φ are the peak width in radial and azimuthal directions. All these parameters were fitted by the least squares method to the experimental intensities. An example of the fit is shown in Fig. S5b. The radial FWHM was calculated as $w_q = 2\sqrt{2\ln 2}\sigma_q$, the azimuthal one as $w_\varphi = 2\sqrt{2\ln 2}\sigma_\varphi q_0$. The parameters were averaged over all peaks of the corresponding Bragg peak family. The errors were estimated as the standard deviation within a Bragg peak family.

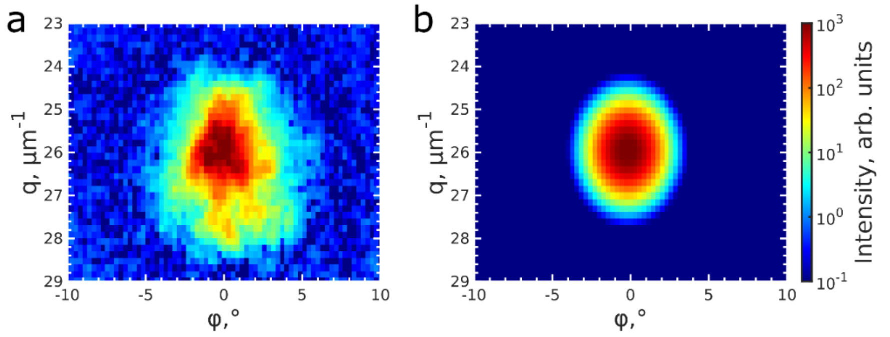

Figure S6. (a) Magnified area in the vicinity of one of the Bragg peaks from the $2\bar{1}\bar{1}0$ family. The peak is highlighted in Fig. S4. **(b)** The result of the peak fitting by a 2D Gaussian function.

S7. Bragg peak fitting while heating

During heating we observed splitting of each Bragg peak into three subpeaks as shown in the main text, Fig. 7e-h. The subpeaks were fitted by a linear combination of three Gaussian functions (eq. S8). Three sets of the parameters were optimized simultaneously. Evolution of the extracted parameters for each subpeak is shown in Fig. S6 – S8.

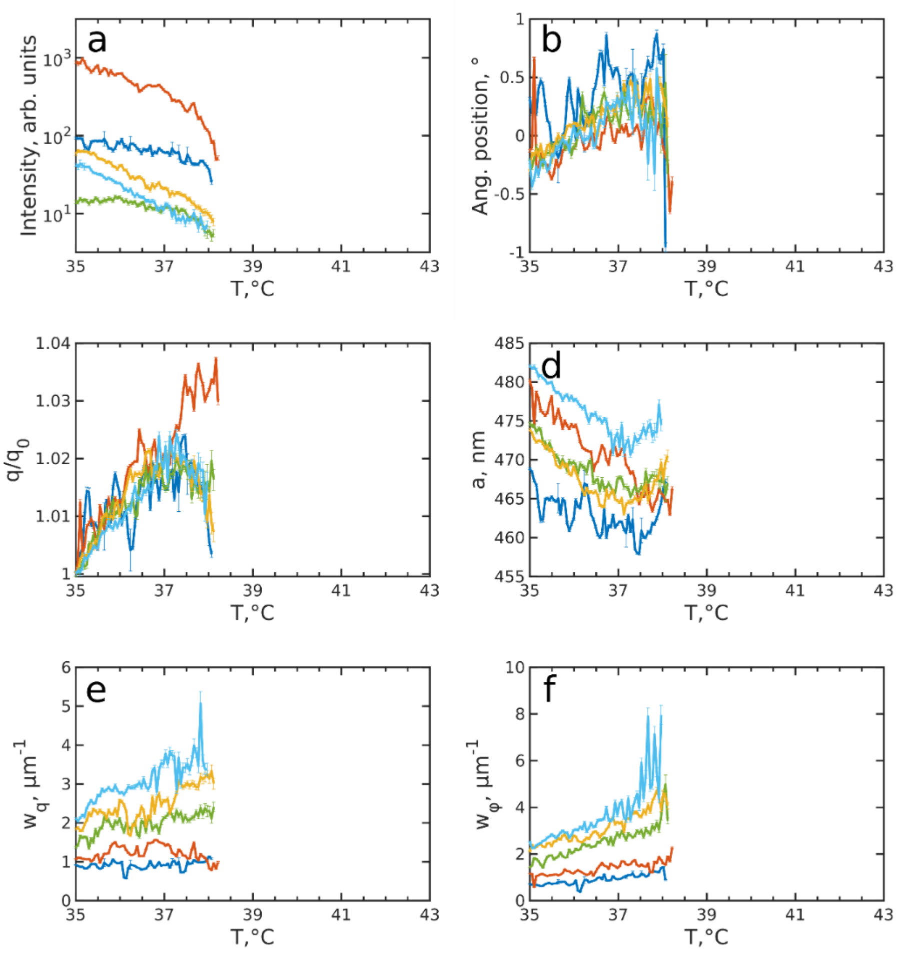

Figure S7. Evolution of the parameters of the “blue” subpeaks of different orders: **(a)** the intensity, **(b)** the angular position, **(c)** the momentum transfer values with respect to the initial values, **(d)** the calculated unit cell parameters, **(e)** the FWHM in radial direction and **(f)** the FWHM in azimuthal direction.

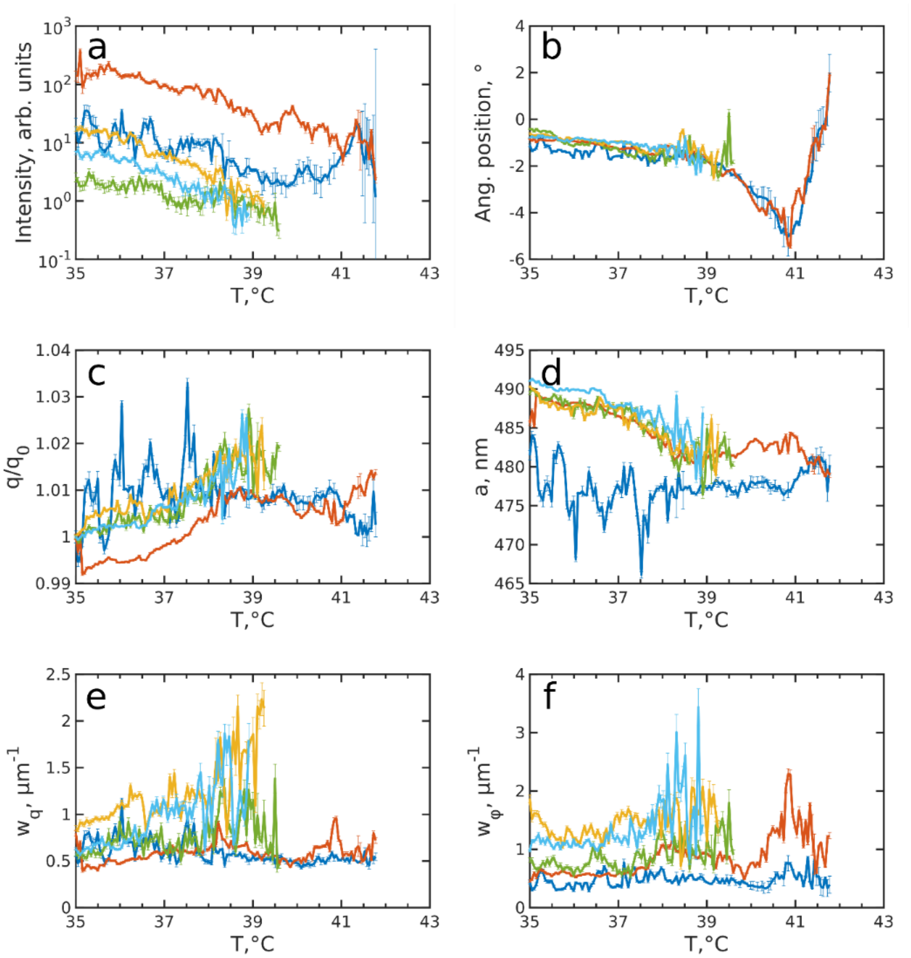

Figure S8. Evolution of the parameters of the "red" subpeaks of different orders: **(a)** the intensity, **(b)** the angular position, **(c)** the momentum transfer values with respect to the initial values, **(d)** the calculated unit cell parameters, **(e)** the FWHM in radial direction and **(f)** the FWHM in azimuthal direction.

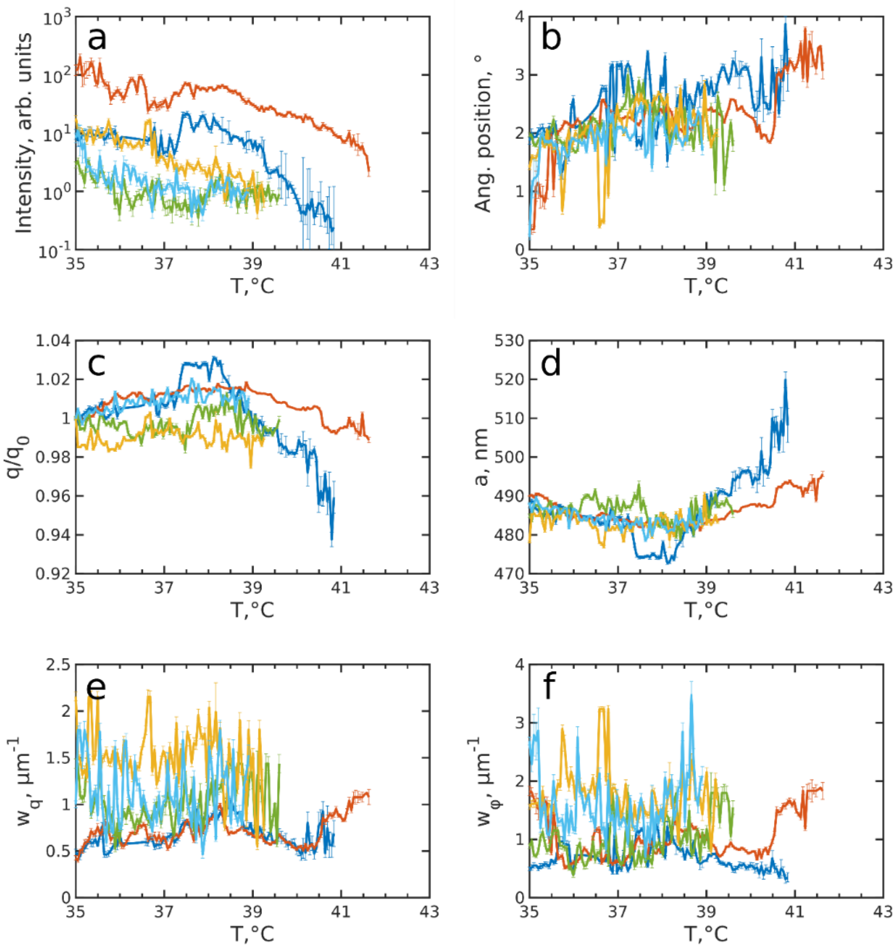

Figure S9. Evolution of the parameters of the "green" subpeaks of different orders: **(a)** the intensity, **(b)** the angular position, **(c)** the momentum transfer values with respect to the initial values, **(d)** the calculated unit cell parameters, **(e)** the FWHM in radial direction and **(f)** the FWHM in azimuthal direction.

Besides the lattice spacing analysis, we performed Williamson-Hall analysis. We did find the results were not reliable because the FWHMs fits contained large errors. However, the resulting distortion values we did obtain for each crystallite are shown in Fig. S9 and again indicate significant difference in the crystallite behaviour. The “blue” crystallite initial distortion values of g_q = 4 ± 1% and g_φ = 5 ± 1% in radial and angular directions, respectively, are very similar to the average values at the end of cooling. During further heating, the distortions monotonically increase and reach the values of g_q = 7 ± 2% and g_φ = 10 ± 2% at T ≈ 38.2 °C, when the peaks disappear, indicating distortions in the crystallite structure grow. For the “red” and “green” crystallites, the values are initially lower at the level of g ≈ 3 ± 1% in both directions and stay almost constant in the whole temperature range until the crystallites fully melt. The latter shows that these crystals do not distort during melting.

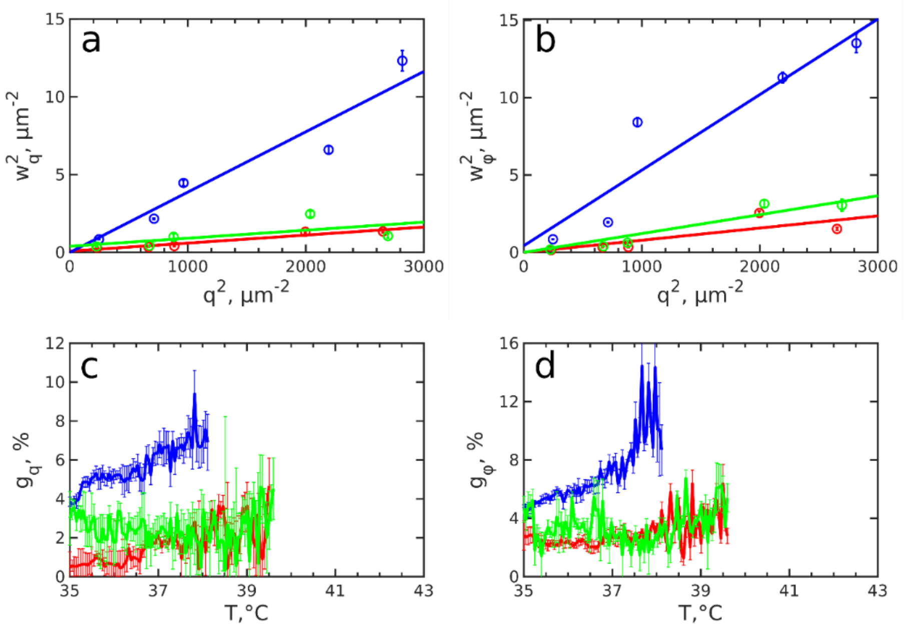

Figure S10. (a,b) Examples of the Williamson-Hall plots for the FWHMs of the subpeaks in (a) radial and (b) azimuthal directions at T = 37.0 °C. Points are experimental values and straight lines are the best fits. **(c,d)** Evolution of the extracted (c) radial and (d) angular lattice distortions during heating.

References:

1. R. Pelton, *Adv. Colloid Interface Sci.,* 2000, **85**, 1-33
2. J. Als-Nielsen and D. McMorrow. *Elements of modern X-ray physics.* John Wiley & Sons, New York (2011)
3. J. S. Pedersen, *Adv. Colloid Interface Sci.,* 1997, **70**, 171-210
4. A. S. A. Mohammed, A. Carino, A. Testino, M. R. Andalibi and A. Cervellino, *J. Appl. Cryst.,* 2019, **52**, 344-350
